# Antibody interference and response kinetics of isatuximab plus pomalidomide and dexamethasone in multiple myeloma

**DOI:** 10.1038/s41408-021-00562-9

**Published:** 2021-10-20

**Authors:** Cyrille Hulin, Meral Beksac, Hugh J. Goodman, Ivan Spicka, Adrian Alegre, Miles Prince, Frank Campana, Greg Finn, Solenn Le-Guennec, Sandrine Macé, Stéphane Muccio, Alexandra Tavernier, Marie-Claude Rouchon, Paul G. Richardson

**Affiliations:** 1 Department of Hematology, Hospital De Haut Leveque, University Hospital, Pessac, France; 2 Ankara University School of Medicine, Department of Hematology, Ankara, Turkey; 3 Waikato Hospital, Hamilton, New Zealand; 4 First Faculty of Medicine, Charles University and General Hospital, First Department of Medicine, Department of Hematology, Prague, Czech Republic; 5 Hematology Service, University Hospital La Princesa, Madrid, Spain; 6 University of Melbourne, Epworth Healthcare, Melbourne, Australia; 7 Sanofi, Cambridge, Mass USA; 8 Sanofi Oncology, Cambridge, MA USA; 9 Sanofi R&D, Vitry-sur-seine, France; 10 Present Address: Sanofi, Montpellier, France

**Keywords:** Tumour immunology, Immunotherapy, Randomized controlled trials

**Dear Editor,**

In multiple myeloma (MM), deep response to treatment is associated with improved progression-free survival (PFS) and overall survival (OS) [1–3]. Furthermore, the depth of response is linked with the long-term outcome of patients with MM [1, 3, 4]. Therefore, attaining a minimal residual disease (MRD) negativity status is one of the most relevant independent prognostic factors in MM [5, 6].

Based on the Phase 3 ICARIA-MM study, isatuximab (Isa, Sarclisa^®^) is approved in a number of countries in combination with pomalidomide and dexamethasone (Pd) for the treatment of adult patients with relapsed/refractory MM (RRMM) who have received ≥2 prior therapies, including lenalidomide and a proteasome inhibitor. Based on the Phase 3 IKEMA study, isatuximab in combination with carfilzomib and dexamethasone is approved in the United States, for the treatment of adult patients with relapsed or refractory MM who have received 1–3 prior lines of therapy, and in the European Union, for the treatment of adult patients with relapsed MM who have received ≥1 prior therapy.

As Isa is an IgG kappa monoclonal antibody (mAb), it may be detected on conventional serum protein electrophoresis (SPEP) and immunofixation electrophoresis (IFE) assays that are used to monitor patients with IgG kappa type M-protein. This interference could lead to false-positive assay results and, an inaccurate determination of a patient’s response to the treatment according to International Myeloma Working Group (IMWG) criteria [7].

This paper reports on both Isa interference with M-protein measurement and depth of response kinetics with Isa-Pd from the ICARIA-MM study.

The ICARIA-MM study (NCT02990338) recruited patients from January 2017 with the last patient last visit in November 2018 as previously described [8]. This study used immuno-capture and liquid chromatography coupled to high-resolution mass spectrometry (IC-LC-HRMS) to evaluate the impact of Isa-mediated M-protein interference on the depth of response of patients treated with Isa-Pd (See supplementary methods). MRD was assessed in bone marrow samples from patients with complete or suspected complete response (CR) by next-generation sequencing at a sensitivity level of 10^−5^ (see Supplementary Methods for further details).

The primary endpoint was PFS, as assessed by an independent response committee (IRC). Key secondary endpoints were overall response rate and OS. PFS and time to response in the intent-to-treat (ITT) population were analyzed using Kaplan–Meier method. Categorical and ordinal data were summarized using the number and percentage of patients in each treatment group. The protocol was approved by independent ethics committees and institutional review boards at all participating institutions prior to the start of the study. Written informed consent was obtained from all participants prior to inclusion in the study. The study was conducted in accordance with the Declaration of Helsinki and the International Conference on Harmonization Guidelines for Good Clinical Practice.

MRD was assessed by next-generation sequencing in bone marrow aspirates (BMAs) from patients who were assumed to have achieved CR by the investigator (prior to IRC confirmation) (Supplementary Fig. 1). BMA samples were collected at baseline, at the time of CR, and if the sample was MRD positive. BMA collection for MRD was repeated 3 months later for late negativity or when clinically indicated. MRD data were obtained from 16 patients (Isa-Pd *n* = 14 and Pd *n* = 2). MRD-negative samples at a sensitivity level of 10^−5^ were detected in 8/14 Isa-Pd patients and 0/2 Pd patients. In an ITT analysis, this results in an MRD negativity rate of 5.2% (*n* = 8/154) with Isa-Pd and 0% (*n* = 0/153) with Pd. Baseline characteristics in patients with MRD negativity are shown in Supplementary Table 1.

There was a correlation between depth of response including MRD negativity and improved long-term outcomes in both Isa-Pd- and Pd-treated patients. All Isa-Pd patients with MRD negativity were still alive and progression-free in the primary analysis at a median follow-up of 11.6 months (Fig. 1A). Within the Isa-Pd arm, median PFS was not reached in the MRD-negative group, whereas median PFS was 15.21 months (13.31–not calculable) in 42 patients who achieved at least a ≥VGPR and were MRD positive (either positive sample or no available sample), 11.53 (8.54–14.78) months in the 44 patients who achieved a partial response (PR) and 3.29 (2.63–4.57) months in the patients not obtaining a response. Similar trends were observed for OS (Fig. 1B). One-year OS rate in the Isa-Pd arm was 100% (95% CI 100–100%) in the MRD-negative group, while it was 92.9% (95% CI 79.5–97.6%) in the patients who achieved at least a very good partial response or better (≥VGPR) and were MRD positive, 82.4% (95% CI 66.4–91.3%) in the patients who achieved PR and 46.4% (95% CI 31.9–59.7%) in patients not obtaining a response.

**Fig. 1:**
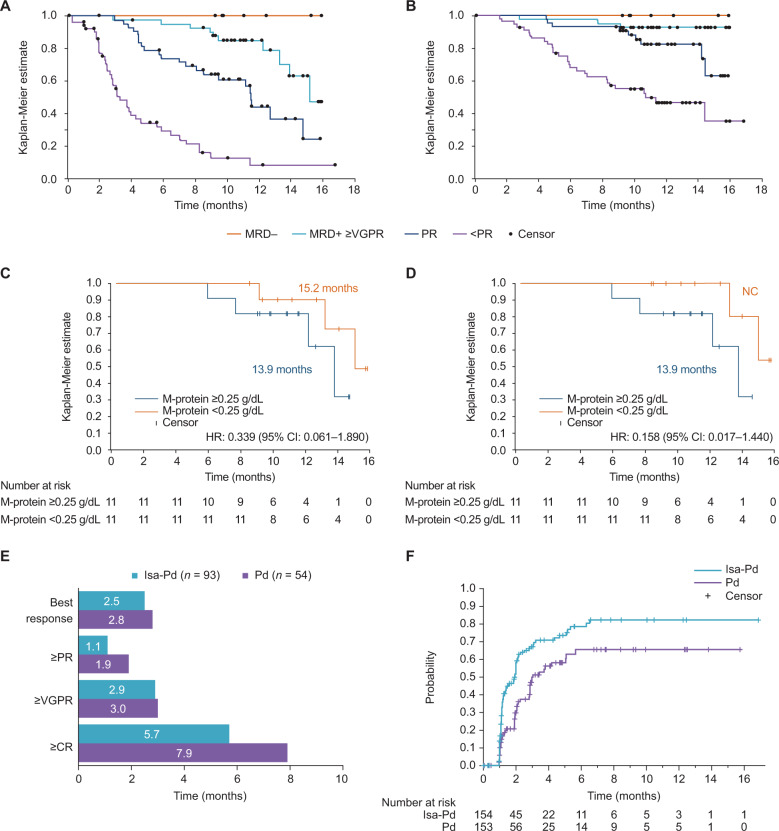
Progression-free survival, overall survival, and time to response. **A** Progression-free survival in the Isa-Pd arm. **B** Overall survival in the Isa-Pd arm by the best overall response and MRD status. **C** Median PFS in months from baseline. **D** TTP in months from baseline per IRC disease assessment for Isa-Pd patients with nCR [1] based on IC-LC-HRMS serum M-protein levels. **E** Time from baseline to best response, first response PR or better, VGPR or better, and CR or better in responders receiving either Isa-Pd or Pd. **F** Time from baseline to first response in patients receiving either Isa-Pd or Pd (ITT population). CI confidence interval, CR complete response, d dexamethasone, HR hazard ratio, IRC independent response committee, Isa isatuximab, ITT intent-to-treat, MRD minimal residual disease, NC not calculable, nCR near complete response, OS overall survival, P pomalidomide, PR partial response, PFS progression-free survival, TTP time to progression, VGPR very good partial response.

Patients receiving Isa-Pd with suspected M-protein interference were selected for mass spectrometry analysis using the near-CR (nCR) criteria [9]. The IRC identified patients with VGPR receiving Isa-Pd who were nCR (defined as a 100% reduction of M-protein by SPEP and less than 5% bone marrow plasma cells while remaining IFE positive). The hypothesis was that for patients meeting all CR criteria except for remaining IFE positive, the IFE signal could be due to the presence of the therapeutic antibody in the serum. Twenty-four patients were identified in the Isa-Pd arm as meeting the nCR criteria. Of those 24 patients, 22 patients had available serum samples for mass spectrometry analysis. The IC-LC-HRMS assay allowed differentiation of Isa and M-protein and thus to overcome the interference of Isa with M-protein measurement observed in conventional IFE assays. Most samples that were below the limit of quantification (1000 µg/mL) in SPEP analysis were quantified using LC-HRMS with a 10 µg/mL equivalent alemtuzumab limit of quantification [10].

M-protein and Isa signals could be separated by IC-LC-HRMS. After separation of the M-protein and the Isa signal, there was no residual M-protein above 250 µg/mL (the threshold for IFE positivity in the study) in 11/22 patients tested, indicating that the true corrected CR rate was underestimated by 7.1%, resulting in a CR rate of 11.7% versus 2.0% for the Isa-Pd versus Pd patients, respectively, due to interference. There was a trend toward longer PFS (Fig. 1C) and time to progression (TTP, Fig. 1D) in patients who would be considered IFE negative based on mass spectrometry versus patients remaining IFE positive. Residual M-protein was above the 10 µg/mL limit of quantification by mass spectrometry and therefore still quantifiable in all cases. Two patients had a progression event.

In the patients obtaining a response, tumors responded faster to Isa-Pd than Pd alone, with a median time to achieving ≥PR of 1 month in the Isa-Pd (*n* = 93) arm versus 1.9 months in the Pd (*n* = 54) arm (Fig. 1E). In the ITT population, the time to first response was also shorter in the Isa-Pd arm than in the Pd arm (Fig. 1F). The median time to first response was 1.94 months (95% CI 1.31–2.00) versus 3.02 months (95% CI 2.83–5.06) in the Isa-Pd versus Pd arms, respectively. The median time to ≥VGPR in the Isa-Pd arm was 10.64 months (range 5.65–NC) and it was not reached in the Pd arm. At 6 months, the cumulative probability of having ≥VGPR was higher in the Isa-Pd arm than in the Pd arm (42.3% [95% CI 32.4–51.9] versus 13.6% [95% CI 7.0–22.4], respectively). Isa efficacy in patients with renal impairment is shown in Supplementary Fig. 2 and detailed in Supplementary Results.

Our study showed that treatment with Isa-Pd resulted in MRD negativity that was striking in patients who had been heavily pretreated and had a poor prognosis. MRD is considered the most meaningful prognostic indicator for a favorable prolonged outcome in patients with newly diagnosed MM [5, 6, 11].

Our study demonstrated that conventional disease assessments (IFE) used in ICARIA-MM resulted in a 7.1% underestimation of CR rate in the Isa-Pd arm in patients with RRMM. This is significant as traditionally, immunoelectrophoresis was adequate in determining urine and serum M-protein responses to therapy in 90% of MM cases [12]. Therapeutic mAbs interfere with routine SPEP and IFA rendering false-positive results for the detection of M-protein [13, 14].

The IC-LC-HRMS analysis confirmed interference mediated by Isa, mostly observed in patients with the IgG isotype and in patients in whom a detectable IgG heavy chain was identified. Interestingly, residual M-protein values were detected by IC-LC-HRMS with the lower range of 18.3 μg/mL still quantifiable above the sensitivity limit of detection of 10 μg/mL. Furthermore, two patients with very low M-protein nadir by IC-LC-HRMS (30 μg/mL and 132 μg/mL) already had a progression event compared with none of the MRD-negative patients. Therefore, future research will need to investigate which method has the most predictive power as a surrogate endpoint.

The kinetics of response were faster in patients receiving Isa-Pd with a median time to first response of 1.9 months compared with 3.0 months in those receiving Pd. Furthermore, a descriptive analysis showed that in patients obtaining a response, tumors responded faster to Isa-Pd than Pd alone with a ≥PR achieved at 1 month versus 1.9 months in the Pd arm.

In conclusion, eight patients in the Isa-Pd arm, including patients with adverse prognostic characteristics, were MRD negative and were progression-free and alive at primary analysis; 1-year OS rate was 100%. Patients who achieved at least a ≥VGPR and were MRD positive had a median PFS of 15.21 months; the 1-year OS rate was 92.9% (95% CI 79.5–97.6%). The depth of response, including MRD negativity, was associated with better long-term survival outcomes, specifically PFS and OS. Mass spectrometry results indicated that interference of Isa with IFE resulted in a 7.1% underestimation of CR in the Isa-Pd arm in patients with RRMM. Importantly, the addition of Isa to Pd in heavily pretreated patients with RRMM resulted in an improved depth of response compared with Pd alone, resulting in more frequent and faster tumor responses, which translates into clinical meaningful implications for real-world practice [15].

## Supplementary information

Supplementary methods and results

## Data Availability

Qualified researchers can request access to patient-level data and related study documents including the clinical study report, study protocol with any amendments, blank case report forms, statistical analysis plan, and dataset specifications. Patient-level data will be anonymized, and study documents will be redacted to protect the privacy of trial participants. Further details on Sanofi’s data-sharing criteria, eligible studies, and process for requesting access are at https://www.clinicalstudydatarequest.com.
