# Supplementary material for: Antibody interference and response kinetics of isatuximab plus pomalidomide and dexamethasone in multiple myeloma
Source: Blood Cancer J. 2021 Oct 20;11(10):169. doi: 10.1038/s41408-021-00562-9 (PMC8528829; doi:10.1038/s41408-021-00562-9)
Supplement: Supplementary file 1 — Supplementary methods and results [file 41408_2021_562_MOESM1_ESM.docx]

**Supplementary methods**

## Isa M-protein interference

Serum immunoglobulins were immuno-captured. Heavy chains (HC) and light chains (LC) were dissociated and sorted using liquid chromatography. M-protein and Isa LC were analyzed using HRMS, which allowed differentiation of the respective accurate mass. As M-proteins are specific for each patient with MM, no standards are available to allow absolute quantification. Therefore, alemtuzumab, (an IgG kappa mAb), was used as a surrogate standard to semi-quantify M-protein in the serum of patients with MM (Supplementary Fig. 1).

**Renal response**

Time to renal response and time to response in responders were summarized using the number of available data, mean, standard deviation, median, minimum, and maximum for each treatment group.

**Supplementary results**

## Efficacy: Renal response

In patients with renal impairment, fast tumor response is important to reverse emerging renal dysfunction. In patients entering the study with renal function impairment (Modification of Diet in Renal Disease formula <50 mL/min/1.73 m^2^ at baseline), complete renal response (CRrenal, defined as <50 mL/min/1.73 m^2^ at baseline and at least one assessment ≥60 mL/min/1.73 m^2^ on treatment) occurred in 23/32 (71.9%) patients in the Isa-Pd arm versus 8/21 (38.1%) patients in the Pd arm; CRrenal was durable (lasting at least 60 days) in 10/32 (31.3%) and 4/21 (19.0%) patients, respectively. Renal responses occurred faster with Isa-Pd than Pd (CRrenal: 3.4 weeks Isa-Pd compared with 7.3 weeks Pd; durable CRrenal 2.4 weeks Isa-Pd group compared with 4.8 weeks Pd group, Supplementary Fig 2).

**Renal Discussion**

Consequently, tumor responses were not only deeper but faster in patients receiving Isa-Pd compared with patients receiving Pd. Fast tumor response in MM is particularly important in a context of organ dysfunction, such as renal function impairment. Among patients with creatinine clearance of <50 mL/min/1.73 m^2^ at baseline, more patients in the Isa-Pd arm showed CRrenal and sustained renal response than those in the Pd arm. In addition, overall progression to severe or end-stage renal disease occurred less frequently in the Isa-Pd arm compared with the Pd arm.

**Supplementary Fig. 1 Description of the immuno-capture and liquid chromatography coupled to high resolution mass spectrometry process.**

**Caption**

1. **Sample process, preparation and analysis.**
2. **Quantification.**


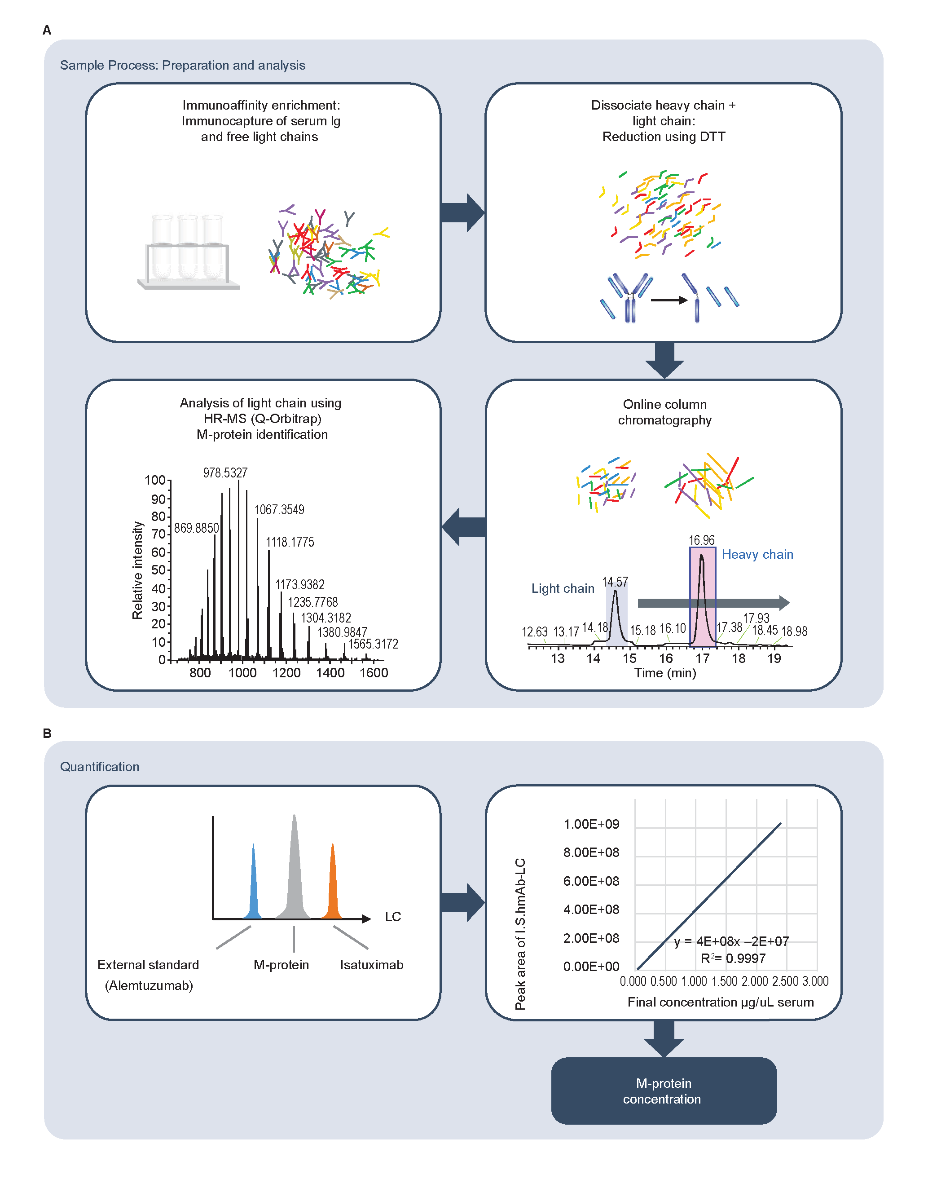


HR-MS, high resolution mass spectrometry; IC, immunocapture; LC, liquid chromatography.

**Supplementary Fig. 2 Renal response**

**Caption**

**Time to CRrenal and durable CRrenal with Isa-Pd and Pd in patients with baseline. eGFR <50 mL/min/1.73 m².**


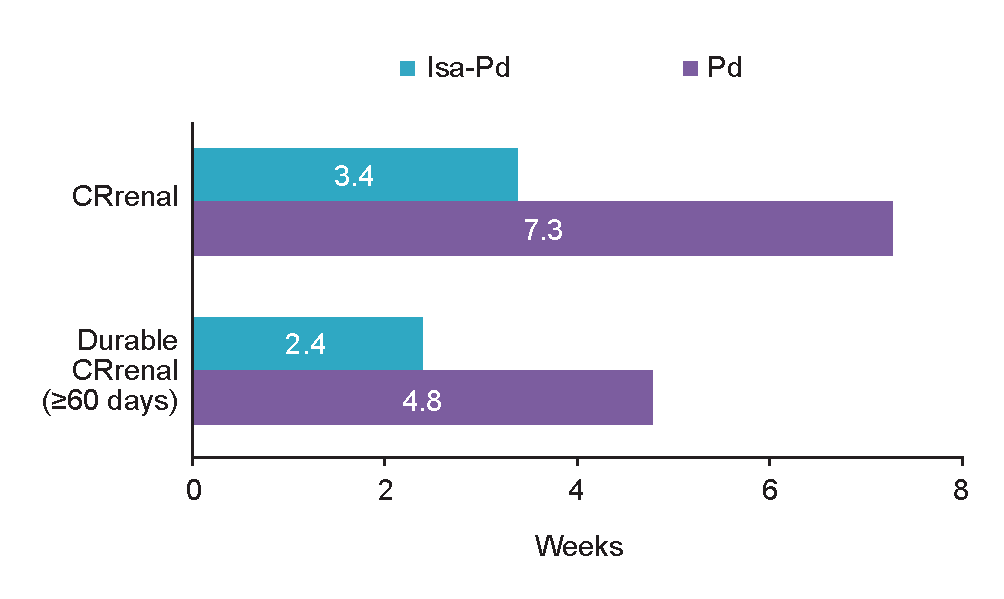


*CRrenal* complete renal response, *d* dexamethasone *eGFR* estimated glomerular filtration rate, *Isa* isatuximab, *P* pomalidomide

**Supplementary Table 1 Demographic characteristics of patients with negative minimal residual disease.**

|  | **MRD negative**  **(*N* = 8)** |
| --- | --- |
| Age (y)  Median  Range | 63.5  54 – 81 |
| Age group, years, n (%)  <65  ≥65–75  >65 | 4 (50.0)  2 (25.0)  2 (25.0) |
| Gender, n (%)  Female  Male | 4 (50.0)  4 (50.0) |
| ECOG performance status, n (%)  0  1  2 | 3 (37.5)  4 (50.0)  1 (12.5) |
| R-ISS stage at study entry, n (%)  Stage I  Stage II | 3 (37.5)  5 (62.5) |
| eGFR (MDRD formula), n (%)  ≥60 to <90 mL/min/1.73 m^2^  ≥45 to <60 mL/min/1.73 m^2^  ≥30 to <45 mL/min/1.73 m^2^ | 3 (37.5)  1 (12.5)  2 (25.0) |
| Number of prior lines, n (%)  2  3  4 | 3 (37.5)  4 (50.0)  1 (12.5) |
| Patients refractory to, n (%)  Lenalidomide  Proteasome inhibitor  Lenalidomide and a proteasome inhibitor | 1 (12.5)  1 (12.5)  1 (12.5) |
| Cytogenetic risk  Standard risk  Unknown or missing | 6 (75.0)  2 (25.0) |

*ECOG* Eastern Cooperative Oncology Group, *eGFR* estimated glomerular filtration rate, *MDRD* Modification of Diet in Renal Disease, *MRD* minimal residual disease, *R-ISS* revised International Staging Score
